# Supplementary material for: Fulfillment, burnout and resilience in emergency medicine—Correlations and effects on patient and provider outcomes
Source: PLoS One. 2020 Oct 19;15(10):e0240934. doi: 10.1371/journal.pone.0240934 (PMC7571699; doi:10.1371/journal.pone.0240934)
Supplement: S1 Table — (DOCX) [file pone.0240934.s001.docx]

S1 Table. Personal Resilience from Healthcare Providers of Different Age Groups

|  | Personal Resilience |
| --- | --- |
| Age Groups |  |
| ≥60 years old | 0.74 (0.46) |
| 50-59 years old | 0.84 (0.48) |
| 40-49 years old | 1.06 (0.60) |
| 30-39 years old | 0.96 (0.53) |
| <30 years old | 0.77 (0.53) |
